# Citizen Panel: Novel method for Public Involvement in data access review of linked longitudinal data

**DOI:** 10.23889/ijpds.v9i5.2774

**Published:** 2024-09-18

**Authors:** Lidis Garbovan, Robin Flaig

**Affiliations:** 1 University of Edinburgh

UK Longitudinal Linkage Collaboration’s (UK LLC) Citizen Panel pilot research project aims to embed public feedback and perceptions into data access decision-making and Trusted Research Environment (TRE) design. The Citizen Panel is a concept developed by Understanding Patient Data as a new methodological approach to decisions about data via conversations with the public.

The Citizen Panel has three key objectives: involving the public in decisions around the acceptance and suitability of data use, assessing the scope and benefit of research questions, and creating a learning feedback loop for decision-making.

The approach taken in the Citizen Panel is intended to enhance and complement the existing ways in which public involvement is conducted in longitudinal studies, especially focused on maximizing diversity and inclusion of seldom-heard communities which are underrepresented in longitudinal studies. The Citizen Panel is conceptualized as a ‘learning model’ which will embed a diverse public voice in planning and decision-making, enabling UK LLC to be responsive to new opportunities and changes in context.

The project will start with a small number (15-20) of public contributors and will explore approaches to scale up in size (panel with >100 members), and geography (e.g. Citizen Panel groups in four UK nations). The project is currently in the design stage (March 2024), which will be followed by recruitment, delivery, evaluation and producing and sharing outputs. The results will be shared widely with academic communities and public networks.

